# Maximum temperatures of 89°C recorded during the mechanical preparation of 35 femoral heads for resurfacing

**DOI:** 10.3109/17453674.2011.636681

**Published:** 2011-11-25

**Authors:** Richard Baker, Michael Whitehouse, Michael Kilshaw, Morreica Pabbruwe, Robert Spencer, Ashley Blom, Gordon Bannister

**Affiliations:** The Avon Orthopaedic Centre, Southmead Hospital, Bristol, UK

## Abstract

**Background and purpose:**

We noticed that our instruments were often too hot to touch after preparing the femoral head for resurfacing, and questioned whether the heat generated could exceed temperatures known to cause osteonecrosis.

**Patients and methods:**

Using an infra-red thermal imaging camera, we measured real-time femoral head temperatures during femoral head reaming in 35 patients undergoing resurfacing hip arthroplasty. 7 patients received an ASR, 8 received a Cormet, and 20 received a Birmingham resurfacing arthroplasty.

**Results:**

The maximum temperature recorded was 89°C. The temperature exceeded 47°C in 28 patients and 70°C in 11. The mean duration of most stages of head preparation was less than 1 min. The mean time exceeded 1 min only on peripheral head reaming of the ASR system. At temperatures lower than 47°C, only 2 femoral heads were exposed long enough to cause osteonecrosis. The highest mean maximum temperatures recorded were 54°C when the proximal femoral head was resected with an oscillating saw and 47°C during peripheral reaming with the crown drill. The modified new Birmingham resurfacing proximal femoral head reamer substantially reduced the maximum temperatures generated. Lavage reduced temperatures to a mean of 18°C.

**Interpretation:**

11 patients were subjected to temperatures sufficient to cause osteonecrosis secondary to thermal insult, regardless of the duration of reaming. In 2 cases only, the length of reaming was long enough to induce damage at lower temperatures. Lavage and sharp instruments should reduce the risk of thermal insult during hip resurfacing.

Hip resurfacing can fail due to osteonecrosis (Amstutz et al. 2004, Daniel et al. 2004). Osteonecrosis has been explored by surrogate means. The femoral head is devascularized by the posterior approach (Steffen et al. 2005, Beaule et al. 2006, Khan et al. 2007) and its blood flow is reduced by 50% if the neck is notched (Beaule et al. 2006). Temperatures during femoral head preparation are unknown and could be a cause of osteonecrosis. Temperatures may reach 68°C when cement is polymerizing during resurfacing (Gill et al. 2007).

The effect of heat generated in bone at the cellular level is difficult to quantify. The important factors are the peak temperature and the duration of the thermal insult. With higher temperatures, a shorter exposure is needed to cause injury (Lundskog 1972, Berman et al. 1984). Thermal insult of 47°C for 60 s is the threshold for bone injury (Ericksson and Albrektsson 1983). Exposure to 50°C for 30 s causes widespread injury to bone 1 mm from the point of exposure (Lundskog 1972) and 55°C for 1 min causes marrow necrosis (Berman et al. 1984). Bone alkaline phosphatase is denatured at 56°C (Posen et al. 1965). When bone reaches to a temperature of 70°C or more, macroscopic bone necrosis can be seen intraoperatively. Cell necrosis occurs at temperatures of 70°C within 1 s (Moritz and Henriques 1947). There is histological evidence of bone necrosis after exposure to 70°C for 1 min (Berman et al. 1984) and 80°C for 5 s (Lundskog 1972).

We noticed that our instruments were often too hot to touch after preparing the femoral head for resurfacing and wondered whether the heat generated during femoral head preparation might exceed the temperatures known to cause osteonecrosis.

## Patients and methods

35 patients undergoing hip resurfacing arthroplasty consented to take part in the study. None of the patients who were approached to enter the study declined. All patients gave informed consent and ethical approval was obtained from the local Research Ethics Committee (reference 07/H0102/71). All patients had a hip resurfacing performed using the manufacturer's standard technique, 8 through a transgluteal approach and 27 through a posterior approach. 5 consultant surgeons performed or supervised the surgery using their normal resurfacing device.

There were 6 female and 29 male patients; their mean age was 51 (23–62) years. No patients had taken or were taking oral corticosteroids. The mean BMI was 28 (21–36). The size of the femoral component was noted in all cases. 2 patients had dysplastic hips with associated osteoarthritic changes; the remainder had idiopathic osteoarthritis. 22 patients had cysts in their femoral head, all of which measured < 1 cm on the preoperative radiographs.

7 patients received an ASR (Depuy International, Leeds, UK), 8 a Cormet (Corin Group PLC, Cirencester, UK), and 20 a Birmingham (Smith & Nephew, London, UK) resurfacing arthroplasty. All the Cormet resurfacings were performed through a transgluteal approach. In the ASR, peripheral reaming, proximal head removal, and chamfering were performed as 1 surgical step. 5 of the patients undergoing a Birmingham hip resurfacing had femoral head preparation performed with the new generation of femoral head reamers. The new generation of Birmingham reamers were available to all surgeons from the start of the study. The main difference in the new reamers is that the proximal part of the head is removed with an end-on cutting reamer rather than the sagittal saw used in the traditional technique, and the instruments were sharper than the much-used originals.

The heat generated during femoral head preparation was measured using an infra-red thermal imaging camera (Thermacam Flir A380 and P95 models; Thermascan, Bedford, UK). The camera was mounted on a tripod opposite the operating surgeon 1.3–1.5 m from the patient. The assistants stood aside to allow an unimpeded view of the femoral head once exposed and dislocated.

The ambient room temperature, distance of the camera from the patient, and emissivity value were entered into the camera before filming. This step calibrates the equipment and allows accurate and reproducible temperature measurement. An emissivity value of 0.99 was used (having been previously calculated on femoral bone in vitro). An emissivity value of 0.99 would lead to a potential error rate of 1% of apparent surface temperatures measured.

The surface temperature of the femoral bone was recorded in real time as the operations were performed. The temperature of the bone was measured adjacent to the edge of the cutting tools. Images were captured every 4 s for the first 8 patients (P95 camera) and every second for patients thereafter (A380 camera). This was due to an upgrade in the camera equipment. Images were captured during the mechanical preparation of the femoral head starting with the first overdrill of the guide-wire, followed by peripheral reaming, proximal head removal, chamfering, and drilling of cement keyholes.

Images were analyzed using Thermacam Pro 2.9 software (Thermascan, Bedford, UK). Images are loaded into the analysis package and any object on the image can have its surface temperature analyzed. The bone temperatures generated were obtained by identifying the area of interest at the bone-instrument interface (Figure), or exposed bone if no cutting instrument was captured in the image.

**Figure:**
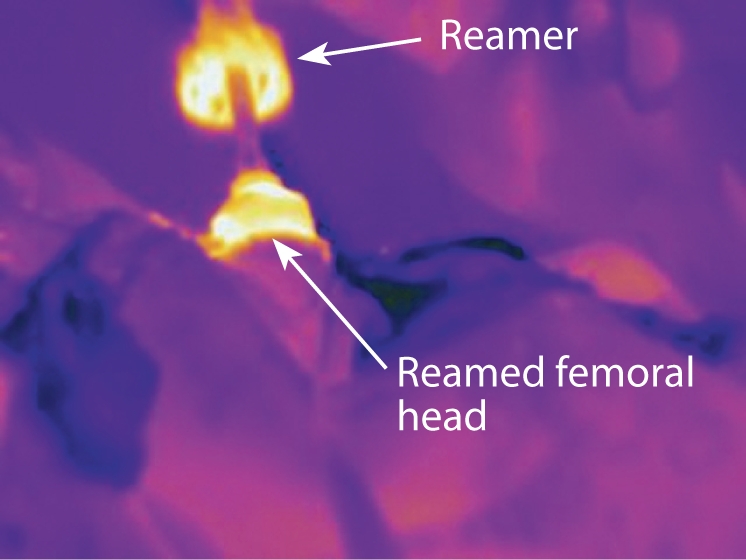
Infra-red thermal image of crown reamer and remaining femoral head during peripheral reaming.

For every image acquired, the maximum temperature of the bone was recorded. For each part of the resurfacing procedure, the maximum temperature achieved was recorded. The mean maximum temperature was calculated by addition of all the maximum temperatures recorded and division by the number of images captured for each step of the surgical procedure.

The time (in seconds) of each surgical step was recorded and the duration of exposure to temperatures exceeding 47°C and 56°C throughout each part of the surgical procedure was noted. Temperatures above 70°C were also recorded, as cellular death will occur regardless of the time of exposure (Moritz and Henriques 1947).

The effect of cooling by pulsatile lavage was measured in 19 patients by recording the minimum bone surface temperatures as the bone was lavaged prior to cementation.

### Statistics

The outcome measure was the temperature generated. The Mann-Whitney test was used for associations involving gender, preoperative cysts, and a postoperative pedestal sign. Pearson's correlation coefficient was used for correlations involving age, femoral component size, and BMI. The Kruksal-Wallis test was used for associations involving type of prosthesis and reaming times; the significance due to prostheses type was obtained using the Mann-Whitney test.

## Results

Data were incomplete during 5 cases of central drilling, and in 1 case chamfering, peripheral head removal and proximal head removal were all obscured by the surgeon. The mean starting temperature of the femoral heads was 26°C (21–30).

None of the femoral necks were notched during surgery. 1 patient, in whom a temperature of 83°C was recorded during proximal head resection, sustained a femoral neck fracture 1 month after surgery. The maximum temperatures generated were not associated with gender, age, body mass index, preoperative cysts, or the size of the femoral component.

### Maximum temperatures (Tables 1 and 2)

11 femoral heads had maximum temperatures of more than 70°C, 7 had maximum temperatures between 56°C and 70°C, 9 between 47°C and 56°C, and 8 had maximum temperatures of 47°C or less during any part of the resurfacing procedure. The highest temperature recorded (89°C) occurred during peripheral reaming during a Cormet resurfacing.

**Table 1.** Temperatures generated during femoral head preparation

Procedure	Over-drilling	Peripheral reaming	Proximal head removal	Chamfer	Key holes	Lavage
Mean temp (range),°C	47 (32–85)	47 (32–89)	54 (33–83)	43 (32–85)	43 (31–62)	22 (18–26)
n	30	34	27	27	21	19
Cases > 47°C	11	14	18	5	6	0
Cases > 56°C	6	5	10	3	3	0
Cases > 70°C	2	3	4	2	0	0

**Table 2.** Maximum temperatures generated by different prosthesis types

Prosthesis (n)	Median maximum temperatures generated (range), °C			
	Over-drilling	Peripheral reaming	Proximal head removal	Chamfer
ASR (7)	46 (32–74)	49 (38–88)	–	–
Birmingham (14)	44 (36–59)	42 (32–56)	56 (35–82)	37 (32–46)
Birmingham New (5)	43 (41–85)	38 (35–56)	38 (32–46)	36 (33–45)
Cormet (8)	40 (32–53) **^a^**	55 (34–89)	55 (46–79)	51 (39–85)

**^a^** (n = 4)

Resection of the proximal head with an oscillating saw caused the highest median temperatures recorded in the Birmingham (56°C) and Cormet resurfacings (55°C). Resection of the proximal head with the new Birmingham created lower mean temperatures than with other prostheses (p = 0.01).

### Duration of femoral head preparation (Table 3)

The mean duration of most stages of head preparation was less than 1 min (median time 53 s). The mean time exceeded 1 min only for peripheral head reaming of the ASR system. This result was skewed by a single patient who underwent 122 s of reaming.

**Table 3.** Time of femoral head preparation

Prosthesis (n)	Median time, s (range)			
	Over-drilling	Peripheral reaming	Proximal head removal	Chamfer
ASR (7)	28 (8–56)	54 (19–122)	–	–
Birmingham (14)	18 (10–52)	22 (7–64)	23 (10–76)	9 (4–32)
Birmingham New (5)	36 (16–74)	20 (6–52)	18 (12–40)	8 (7–10)
Cormet (8)	21 (20–26) **^a^**	36 (21–155)	21 (10–36)	28 (6–69)

**^a^** (n = 4)

### Duration of femoral head preparation and temperature (Table 4)

At the lower temperatures of 47°C and 56°C, no femoral heads were exposed long enough (i.e. more than 60 s) to cause damage during central drilling, proximal head removal, or chamfering. However, during peripheral femoral head reaming 1 patient was exposed to 90 s of reaming at > 47°C (74 s of which were > 56°C with an ASR) and another patient was exposed to 107 s of reaming at > 47°C (54 s of which were > 56°C with a Cormet). Both of these patients also had bone temperatures in excess of 70°C. Therefore, at lower temperatures, only 2 femoral heads were exposed long enough to cause osteonecrosis.

**Table 4.** Time femoral head exposed to temperatures > 47°C and > 56°C

Prosthesis (n)	Median time, s (range)							
	Over-drilling		Peripheral reaming		Proximal head removal		Chamfer	
	> 47 °C	> 56 °C	> 47°C	> 56°C	> 47 °C	> 56 °C	> 47 °C	> 56 °C
ASR	14 (10–18)	2 (2–8)	16 (2–90)	37 (2–72)	–	–	–	–
Birmingham	7 (2–12)	5 (1–12)	4 (3–11)	1	8 (2–15)	4 (1–6)	–	–
Birmingham New	12 (2–22)	6	2	–	–	–	–	–
Cormet	–	–	16 (2–107)	9 (4–54)	14 (2–28)	6 (2–14)	25 (5–35)	18 (13–20)

The Cormet chamfer exposed the bone to temperatures greater than 47°C (p = 0.002) and greater than 56°C (p = 0.01) longer than the Birmingham systems. The Cormet chamfer reamer was the only one to generate temperatures greater than 47°C.

### Key holes and pulsed lavage

Key-hole temperatures were only recorded in 21 patients. The mean temperature of the bone surface during key-hole drilling was 43°C (31–62). 6 patients had temperatures greater than 47°C, and 3 had temperatures greater than 56°C, which was brief and only captured on single frames of the sequence. It was not possible to measure the time of drilling due to the intermittent and quick nature of the work during this stage of the procedure.

Pulsed lavage with normal saline at room temperature cooled femoral heads by a mean of 4°C (0–10) before cementing.

## Discussion

We believe that this is the first study to document the temperatures generated during hip femoral head preparation for hip resurfacing without the use of thermocouples, which are prone to movement and damage during femoral head preparation.

The temperatures generated during hip resurfacing were great enough to cause thermal damage in 11 patients, regardless of the duration of preparation. 2 of these patients were also subjected to lower temperatures for a duration that was long enough to cause osteonecrosis (due to protracted reaming on hard bone with blunt reamers). More modern reaming systems are sharper and ream bone more quickly—and at lower temperatures than earlier designs.

Blunt drills increase the amount of heat generated (Matthews and Hirsch 1972). Water coolant with high-speed drills reduces bone damage in oral surgery (Costich et al. 1964, Moss 1964). Davidson and James (2003) found that when drilling cortical bone, the temperature did not exceed 50°C when a water coolant was used.

The main limitation of our study was that we were only able to measure the surface temperature of the bone during resurfacing and could not estimate the internal temperature of the remaining femoral head. Berman et al. (1984) showed a 1.5-mm depth of injury when bone was subjected to a scald temperature of 85°C, and an injury of this depth would affect the cement-bone interface.

The temperatures generated during head preparation in hip resurfacing may contribute to bone necrosis, along with the heat generated during cementation of the femoral component (Gill et al. 2007, Hsieh et al. 2008, Little et al. 2008).

This observational study was too limited for us to draw conclusions about specific patient variables that may contribute to temperature differences by the small number of patients studied. However, despite this, all reamer types generated temperatures high enough to cause thermal damage to the remaining femoral bone. The effects of blood flow, force of reaming, surgeon variability, and bone quality are additional variables that were not assessed, as we wanted to observe the temperatures generated in our contemporary practice. The small number of female patients enrolled was due to a decrease in the use of resurfacing in this patient population, due to the recent concerns about pseudotumors (Pandit et al. 2008).

We did not determine the effect of thermal damage during femoral head preparation. However, the damage caused by femoral head preparation, the exothermic reaction of bone cement, and potential devascularization by the posterior approach represent a “triple insult” to the remaining bone of the femoral head during hip resurfacing.

The maximum temperatures generated were high enough to induce cellular necrosis, but the duration of femoral head preparation was short and it is unlikely that thermal injury would occur at lower temperatures. However, cooling of the femoral head is simple to achieve intraoperatively, reduces the maximum temperatures generated, and could help protect the remaining bone from thermal damage. Sharp surgical instruments should also reduce thermal damage because they reduce the length of head preparation, and regular sharpening of older instruments is good practice.
